# Health Systems in High-Performance Sport: Key Functions to Protect Health and Optimize Performance in Elite Athletes

**DOI:** 10.1007/s40279-023-01855-8

**Published:** 2023-06-07

**Authors:** Michael K. Drew, Liam A. Toohey, Miriam Smith, Christine M. Baugh, Hannah Carter, Steven M. McPhail, Jenny Jacobsson, Toomas Timpka, Renee Appaneal

**Affiliations:** 1 Australian Institute of Sport, Bruce, Australia; 2 University of Canberra Research Institute for Sport and Exercise (UCRISE), Canberra, Australia; 3 Division of General Internal Medicine, University of Colorado School of Medicine, Aurora, CO USA; 4 Center for Bioethics and Humanities, University of Colorado, Aurora, CO USA; 5 Australian Centre for Health Services Innovation and Centre for Healthcare Transformation, School of Public Health and Social Work, Queensland University of Technology, Brisbane, Australia; 6 Digital Health and Informatics Directorate, Metro South Health, Brisbane, Australia; 7 Athletics Research Center, Linköping University, Linköping, Sweden; 8 Department of Health, Medicine and Caring Sciences, Division of Society and Health, Linköping University, Linköping, Sweden; 9 Center for Health Services Development, Region Östergötland, Linköping, Sweden; 10 University of Canberra Research Institute for Sport and Exercise (UCRISE), Bruce, ACT 2617 Australia

## Abstract

Enabling athletes to achieve peak performances while also maintaining high levels of health is contextually complex. We aim to describe what a ‘health system’ is and apply the essential functions of stewardship, financing, provision of services and resource generation to an Australian high-performance sport context. We introduce a fifth function that health systems should not detract from athletes’ ability to achieve their sports goals. We describe how these functions aim to achieve four overall outcomes of safeguarding the health of the athletes, responding to expectations, providing financial and social protection against the costs of ill health, and efficient use of resources. Lastly, we conclude with key challenges and potential solutions for developing an integrated health system within the overall performance system in high-performance sport.

## Key Points

**Table:** 

A ‘health system’ is defined as all organizations, people and actions whose primary intent is to promote, restore or maintain health.
Many sport science and medical services aim to promote, restore and maintain health and therefore can be considered within a ‘health system’, yet little attention has been paid to how these services may be understood, optimized and governed.

## Introduction

Enabling athletes to achieve peak performances near the limits of human capability while also maintaining high levels of physical and mental health is contextually complex. We propose that a health system perspective is overdue in high-performance sport. We start by defining what a health system is and provide justification for it based on recent events. We describe sport health services using exemplar legislation and discuss funding as well as trends towards value-based care. We conclude with key challenges and potential solutions for developing an integrated health system within the overarching performance system [1, 2] in high-performance sport.

Adopting a health systems approach provides an integrated framework for prevention and management of athlete health problems, reduces the implementation gap for adopting evidence-based programmes, and enables an evaluation of the health system, which often involves multiple co-interventions with a combined effect upon the population health across sports. We recommend that sport governing bodies adopt this approach to evaluate the performance of their health systems and develop policies which enable health systems in sport to improve, restore and maintain athletes’ health to achieve sustainable sports success. Throughout this paper, we use Australian high-performance sport to illustrate generalizable principles of a health system within a sport environment, with comparisons with other sport environments in different nations.

## A Health Systems Perspective on High-Performance Sport

How health relates to performance [1, 3] and how sports medicine professionals contribute to performance outcomes [2, 4–6] is frequently debated [7, 8]. Furthermore, the leadership and communication styles of coaches can influence health, indicating a bidirectional relationship between health and performance systems [9, 10]. For this paper, we have adapted the World Health Organization (WHO) definition of health to define health in high-performance sport as ‘a state of optimal physical, mental and social wellbeing related to an athlete's sports success, and not merely the absence of illness or injury that limits participation’. Simply stated, the health of an elite athlete provides them with the capacity to pursue her or his life goals, with this capacity including mental, physical and social aspects as well as quality of life upon retirement from sport. The principal task of health services is to safeguard this capacity, and ultimately informs how health systems in high-performance sport are evaluated. There are several performance indicators to consider that link athlete health to sport performance outcomes [3]. These include long-term athlete or team availability rates prior to and during competition, team or individual injury incidence, health status at the point of competition, and prevalence of health problems within a population [3]. A comprehensive overview of health system performance indicators is beyond the scope of this paper.

Beyond the obvious benefits of health for sport performance, increased public attention on recent and historical events in sport underscores the urgent need for a system to safeguard the health and welfare of athletes. Specifically, investigations of athlete mistreatment and abuse have occurred in Australia [11], Japan [12], New Zealand [13], the USA [14] and the UK [15] which have triggered calls for immediate change in sport culture [16–18]. These events highlight the importance of health stewardship to protect against a ‘win-at-all-costs’ culture and to mitigate the unacceptable risk to the safety and wellbeing of young athletes [11]. Failing to create systems and a culture that allow injuries to be disclosed to the sport by the athlete could be considered a form of abuse [13]. The evidence on the association between health and performance outcomes combined with safety and welfare concerns reinforce the need to prioritize athlete health in all sport environments.

## What is a ‘Health System’ and a ‘Health Service’?

The WHO defines a health system as ‘all organizations, people and actions whose primary intent is to promote, restore or maintain health’. [19, 20] A health system is a societal entity that provides health services to its members. A ‘good health system’ has been described as a health system that ‘delivers quality services to all people, when and where they need them. The exact configuration of services varies from country to country, but in all cases requires a robust financing mechanism; a well-trained and adequately paid workforce; reliable information on which to base decisions and policies; well-maintained facilities and logistics to deliver quality medicines and technologies.’ [21] To interpret this, it is important to review how a ‘health activity’ or ‘health service’ is defined. Formal health services, including professional delivery of medical attention, are clearly within this boundary. A useful definition of a health service in Australia is [22]:

1. An activity performed in relation to an individual is a health service if the activity is intended or claimed (expressly or otherwise) by the individual or the person performing it:
  - to assess, maintain or improve the individual’s health; or
  - where the individual’s health cannot be maintained or improved—to manage the individual’s health; or
  - to diagnose the individual's illness, disability or injury; or
  - to treat the individual’s illness, disability or injury or suspected illness, disability or injury; or (e) to record the individual’s health for the purposes of assessing, maintaining, improving or managing the individual’s health.
2. The dispensing on prescription of a drug or medicinal preparation by a pharmacist is a health service.
3. To avoid doubt:
  - a reference in this section to an individual’s health includes the individual’s physical or psychological health; and
  - an activity mentioned in subsection (1) or (2) that takes place in the course of providing aged care, palliative care or care for a person with a disability is a health service.
4. The regulations may prescribe an activity that, despite subsections (1) and (2) is not to be treated as a health service for the purposes of this Act.

Importantly, many services provided to an athlete are typically considered health services, whether labelled ‘performance services’, ‘health services’, ‘clinical services’ or more broadly the practice of sport science when it relates to assessing, predicting, maintaining or improving an athlete’s physical, mental or psychological health or status. Examples may include physiological assessments, nutrition and healthy lifestyle advice, sleep assessments, training stress (training load) applied to athletes, psychological assessment and interventions in addition to traditional medical and allied health services related to the diagnosis and management of injury and illness.

## Requirements of a Health System for High-Performance Sport

According to the WHO, a formal health system should fulfill four fundamental requirements; (1) safeguard the health of the population it serves (including addressing inequity), (2) respond to people’s expectations, (3) provide individuals financial protection against the costs of ill health and (4) be efficient [23]. Importantly, health systems should fulfill these requirements while treating individuals with dignity [24]. All four requirements of a health system are directly applicable to the high-performance sports context. Where they are not met, decreased satisfaction can be expected to occur with wide-ranging implications [23, 25].

High-performance sport adds complexity in that the performance of the health system may be judged against other outcomes, including whether an athlete wins or achieves their goals [2, 8]. To fulfil these requirements, health systems must provide services, generate human and physical resources that make service delivery possible by raising and pooling the resources used to pay for health care, and provide the function of stewardship [23]. In high-performance sport, there is an expectation that these activities support performance achievements. Therefore, it could be argued in this context that a fifth requirement on a health system is to support, and not detract from, sports performance goals. It should be noted, however, that the tension between athlete health and services to increase performance has been discussed at the highest levels of governments around the world during the last decade, including the Australian Senate [26], resulting in greater attention to sport integrity including the formation of Sport Integrity Australia, a government agency to oversee such issues and complaints.

## Configuration of a Health System for High-Performance Sport

Based upon WHO recommendations for implementation of the four essential functions [23] of a health system, we propose that health systems within high-performance sport should equally aim to provide these. Investigating the functions of a health system and how they combine poses an opportunity to evaluate the performance of what the system does rather than the more simplistic view of what it provides.

### Stewardship

Stewardship in health refers to the wide range of functions carried out by governing bodies as they seek to achieve health policy objectives [27]. Stewardship is not a new concept in public policy. While attributes are practiced in high-performance sports contexts, the concept of stewardship has not been fully explored. Stewardship has been identified as the most important function of a health system—and that without it, the other functions are futile [28]. Stewardship may be further broken down into five core attributes: (1) responsible manager, (2) political will, (3) normative dimension, (4) balance interventionist and (5) proponents of good governance [28]. Within high-performance sport, examples of stewardship include requirements for Australian National Sports Organisations to undertake wellbeing checks [29] and rule changes in relation to concussion to improve athlete safety by International Sports Federations and National Sports Organisations [30], as well as the development of a safety policy for serious injury in Rugby Australia [31].

Stewardship may be achieved through three key objectives: (1) setting, implementing and monitoring the rules for the health system; (2) assuring a level playing field for all actors in the system (particularly purchasers, providers and patients) and (3) defining strategic directions for the health system as a whole. To achieve these, a governing body can intervene via several mechanisms. First and foremost, the organization may influence the overall system design. This relates to the policy formulation at the highest level and deals with the organization and integration of all functions of the health system. Examples in sport include the need for a first-aid officer or physiotherapist to be present at a community-level game or an independent concussion medical officer in professional rugby union [30]. There is emerging evidence that the overall system and structures influence the health outcomes of athletes [32–34]. For instance, when medical staff are organizationally required to report to athletic departments (rather than a health services department) in the National Collegiate Athletic Association (NCAA), there is a higher rate of injury recorded [34]. Further aspects are the action of undertaking performance assessments of the subcomponents of the health system, and providing advice for improvements relating to:

- revenue collection, purchasing, provision and resource development;
- priority setting, by choosing criteria for setting priorities and then building consensus among stakeholders, partners and between jurisdictions—a function that has been well characterized in prevention and implementation models over the last three decades [35–37];
- intersectoral advocacy, promotion of policies in other systems that will advance health goals [38];
- setting and overseeing regulation by setting rules and ensuring compliance; and
- consumer protection, achieved by addressing the asymmetry of power between patients and providers to ensure an even playing field for those working within the health system and the consumers of their services.

### Financing

Health system funding is the process by which revenue is collected by primary and secondary sources [23]. Three financing functions include revenue collection, fund pooling and purchasing. In a non-sport setting, evidence suggests that the manner in which health care is organized and funded affects equity and quality of care [39, 40]. In the sports sector, emerging evidence suggests the financial and reporting structure influences health outcomes [33, 34]. Within NCAA institutions, for example, sports medicine services housed within athletics departments reported 31% higher injury incidence rates than those which were external to athletics departments (e.g. student health) [34]. Requirements for athletes to self-fund health care, which may be beyond their financial means, [25] highlight that financial structures in a sports health system should also be considered when analysing health outcomes.

The financing of national health systems varies considerably between countries and may directly impact on the systems adopted within sport [25]. Broadly, Australia has a hybrid public/private health care system. Universal health coverage is available to all Australians under the Medicare programme, which is jointly funded by Federal and State governments. This system provides patients with free access to services at public hospitals and hospital-based providers, with relatively small co-payments required for pharmaceuticals and some primary care services. Australians may also decide to purchase private health insurance that provides access to private hospitals and additional services that may be limited to high-needs patients within the public system, such as dental care and allied health services [41]. Financing of the Australian high-performance sport health system integrates with the general Australian health system for provision of some services (e.g. hospital admissions) but also has a separately funded system dedicated to maintaining the ongoing health and wellbeing of athletes.

#### Revenue Collection

Revenue collection involves the collection of financial resources from primary sources (e.g. individuals) and secondary sources (e.g. governments and donors). There are many factors that influence the mechanism of funding; however, at the core is ensuring adequate funding is available and reducing replication of organizations overseeing and administering the funding. Within a sports setting, primary sources such as out-of-pocket expenses are present as evidenced by athlete co-investment into their health services within the (Australian) Olympic sector and limited co-investment required within the professional sport sector. To date, there are no reports available to indicate to what level these out-of-pocket costs are incurred by athletes, and no reports of expenditure by National Sports Organisations (NSOs)/professional clubs to provide or subsidise health services. Across nations, jurisdictions, sports and sports programmes it is likely to vary greatly.

Secondary sources of revenue collection are typically provided by federal or state government grants administered by the relevant NSO, private or corporate sponsorship to the NSO or club, and benevolent donations. Secondary sources of income provide investment into the organization, and from there it is the decision of sport management to determine how resources are allocated across the health system.

### Fund Pooling

Fund pooling is the accumulation of revenues for the common advantage, typically due to the wider distribution of financial risk, and the ability to gain efficiencies through economies of scale. There are several considerations in pooling of funds which are beyond the scope of this paper. Murray and Frenk (2000) provide an overview of these considerations as they relate to public health systems [23]. Within the Australian high-performance sport environment, there is an ability to pool funding and resources across federal and state jurisdictions. For example, a NSO can pool funds with one or more state or national sports institutes [42]. In other countries and sports organisations, it is common for funds to be pooled across sports programmes to provide health services, such as within an NCAA setting where athletes access health services through an athletic or sports medicine clinic which is mutually funded by one or more sport programmes. Fund pooling is particularly useful when resources are scarce or where health service demand does not warrant an isolated investment by a single sport or sports organization.

#### Purchasing

Purchasing is the process through which revenues collected are allocated to institutions or individual providers. This requires strategic design to ensure the quantum and quality of service provision is adequate to the needs of the population. Purchasing can be direct (specific interventions such as immunisations), general purchasing (e.g. physician services, protective equipment, etc.) and purchasing inputs (e.g. doctors, vehicles, clinical areas, etc.).

Within health care purchasing globally, there is an increasing recognition of the importance of value-based care [43]. High-value care is simply care that provides a high level of health benefit relative to the resources required to deliver it [44]. This reflects broader concerns around the financial sustainability of health care systems, given the increasing levels of demand being driven by ageing populations and greater prevalence of chronic disease. Rigorous, evidence-based health technology assessment processes have been adopted by health care funders in many countries including Australia, the UK and Canada to ensure that all new treatments are safe, effective and cost effective [45–48]. These processes allow for resources to be used more efficiently, so that health benefits can be maximized within the given level of funding available.

To achieve high-value care, important aspects of purchasing strategy may include the choice of providers, the level of control over these providers and the payment mechanism. This may also extend to how health care providers are reimbursed, including via salary, capitation or fee-for-service models. Beyond value considerations, the manner of engaging service providers has been found to impact equity and quality of care in public health systems [40]. Further research is required to understand the impact of alternative purchasing strategies and payment mechanisms within a sports context.

#### Insurance and Other Mechanisms of Financing

Insurance is another option that may be used to fund health and medical services. In New Zealand, the Accident Compensation Corporation monitors sport, traffic and work injuries as a distinct segment of the health care system [49]. Compulsory insurance, as a component of player registration, subsidises health care costs arising from sports injuries in Australia. Each Australian NSO provides a level of cover to their registered participants, which varies in terms and conditions. However, it has been suggested Australia would benefit from a universal no fault insurance scheme in line with the New Zealand model [49] which differs from the current sports insurance mechanisms provided by individual NSOs to their participants.

Individual athletes’ private health insurance is also utilized to subsidise health expenditure. This allows for subsidised access to services such as physiotherapy, nutrition, remedial massage, dental care and in some cases alternative medicine services such as acupuncture [50]. However, the level of subsidy is likely below the needs of an athlete. For instance, in Australia physiotherapy is considered ‘extra’ cover [51], with insurers typically providing either a percentage of the fee per consultation (e.g. 50%) or a set rate per consultation (e.g. AU$42 per initial and AU$30 for a follow-up appointment) until the annual level is reached. An athlete in both arrangements is likely to need to co-pay and will soon exceed this annual level when injured.

### Service Provision

**Fig. 1:**
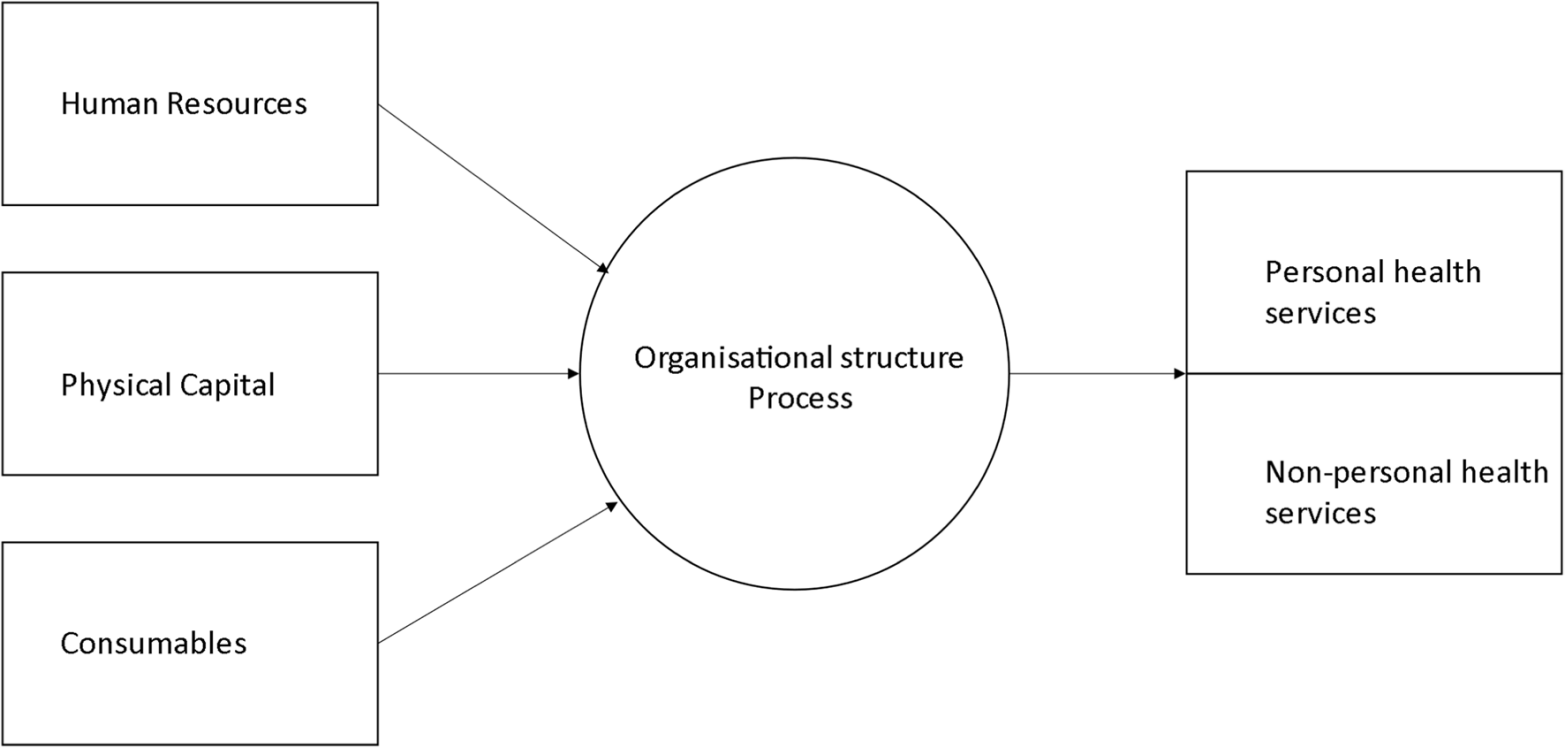
Health service provision. Adapted from Adams et al. [52], with permission

Service provision can be broadly divided into personal and population (non-personal) health services. The level of integration of these services underscores the performance of the health system. Personal services refer to services consumed by the individual, whether these be preventive, diagnostic, therapeutic or rehabilitative, and whether or not they generate externalities [23]. Furthermore, under the definition of health service in Australia, most sport science and sport medicine professions are considered a health service when claiming to ‘assess, predict, maintain or improve the individual’s physical, mental or psychological health or status’ [24]. Population, or ‘non-personal’, services relate to activities that are applied to collectives (e.g. mass health education) or non-human components of the environment (e.g. basic sanitation, build environment, etc.) [23] Fig. 1 schematically represents the inputs and throughputs related to delivering personal and non-personal health services [52, 53]. Both service provision types can be applied to the high-performance sport environment. Personal health services include the provision of rehabilitation to an injured athlete, on-field immediate care of a concussed athlete, surgery provided to an athlete with a ruptured anterior cruciate ligament (ACL) and assessing the cardiovascular fitness of an athlete via a V0_2max_ test. Population health services reflect system or organization-wide initiatives, such as the ‘Football Australia Perform+’ programme by Football Australia [54], a modified version of the FIFA 11+ injury prevention programme [55], provided to community-level football players, or nutritional information/education to improve recovery and adaptation to training.

Within Australian Olympic sport, there are two main providers of health services, one public and the other within the sport system itself. Health services delivered within the sport system include the NSO or National Institute Network, which consists of state and national sports institutes and academies [56]). The Australian public system includes primary care services such as general practitioners, public health programmes, hospitals and private providers (e.g., physiotherapy, nutrition, psychology, podiatry, etc.). Therefore, the sport health system is a complex mix of public, private and organizational service delivery. Health services provided by a health service provider in an unregulated profession are also included. This includes massage therapists, traditional healers and other forms of unregulated therapists (e.g. iridologists, naturopaths), and all medication, whether prescribed by a provider or not. Public health activities such as health promotion and disease prevention (such as vaccinations), and other health-improving interventions such as road safety and built environmental improvements (e.g. playing surfaces) may also be considered within the sports health system; however, these are administered by other government agencies in which sportspeople are not the primary/sole beneficiary.

At the individual care level, access and the mix of skillsets influence the performance of the health system [23]. To date, there is limited evidence for any operating model of care in high-performance sport. UK Athletics published their integrated care model following the 2012 London Olympics and Paralympic Games [2] which outlined an integrated approach of managing between coaching and medical staff and presented an example of how this can be implemented. While comprehensive in its design, it does not cover all features of a health system. The same would be true in the Australian sports health system when considered in isolation from the Australian health system. The Australian Institute of Sport produced a statement, ‘Proactive Model of Care: Focus on Athlete Availability’ in 2017 [57] which detailed the various ways and functions (tasks) service providers (physiotherapists, strength and conditioning coaches, etc.) may provide within a sport health system while providing suggestions for improvements.

Conceptually, many of the same issues raised in personal health services exist in population health services. The key question for population health services is who provides what service. In public health, it is important to understand whether one organization provides services or whether multiple specialized organizations provide these. Following this key question, the next is the level in which these services, such as health promotion, are integrated across the health system. These questions are also relevant for a high-performance sport system as the extent of integration with purchasing is important, as are the level of autonomy and the overseeing governance of any services provided.

Population health services also entail the wider environment, and as such the provision of a safe training and competition environment becomes an element of the health system. For example, a playing field with rocks, sharp objects or divots is likely to lead to injury in a contact sport such as rugby. The provision of a clean competition environment is considered a non-personal health service, such as ensuring water quality is safe in aquatic sports (i.e. open water swimming, triathlon, sailing and rowing) [58, 59]. In both examples, it is the responsibility of the host or governing body to provide safe environment as a component of a population health service.

### Resource Generation

Health systems are not limited to organizations and institutions that fund or deliver services. They also include organizations that provide inputs to the system, with emphasis on human resources, physical resources and knowledge [23, 60]. This includes universities, other education institutions and research centres who provide both training and competition facilities, new knowledge, programme delivery and the development of physical resources such as information booklets related to health and safety in sport. Furthermore, resources may also be generated by companies who provide products such as pharmaceutical products (including medications), nutritional supplements, devices and equipment [23].

The organizations involved in resource generation determine the strategic design, structural arrangements and implementation of health services [23]. In the Australian high-performance sport health system, this is seen in the federated model of government, with each state and territory providing a greater or lesser amount of resource to any given NSO for the provision of health services. This resourcing arrangement introduces issues relating to who is the primary organization, i.e. whether they ‘belong’ to the NSO or NIN. At a government level, key issues also relate to the primary ministry, i.e. do they ‘belong’ to the Department of Health or the Department of Sport. In Australia, the Department of Sport is integrated into the Department of Health, though having a separate Minister.

In the provision of equipment and devices, questions of priority, effectiveness and autonomy become salient, particularly due to the interests of the company providing the device. In high-performance sport, new technology is frequently marketed to reduce injury and key questions to the health system relate to the cost-to-benefit ratio, opportunity costs related to investment (is the money better spent elsewhere?), the independence and external validity of the claims for effectiveness in their environment, human resources to implement the technology and the skills of the user to interpret any information generated by the device. These factors may prohibit the uptake of new technology and devices by the health system when they are not addressed.

### A Health System to Support, and Not Detract From, Sports Performance Outcomes in Elite Athlete Settings

The overarching goal of a high-performance sport system is to support athletes to reach their desired performance outcomes. However, we mark out the safeguarding of athlete health to be the principal task of health services. This tension requires balance, clarity, greater exploration of ‘Are clinicians primarily the athlete’s advocates or are they in the service “of the high-performance sport system”’? That is, should they always prioritize the athlete’s health or are there situations in which they should consider the outcome for the team or nation? How is safeguarding to be balanced against performance in practice? These questions are outside the scope of this review and we draw attention to prior publications on the topic of medical ethics in sport [2, 61–64]. To have an informed debate about goals, there is a need to differentiate between goals that are valued within themselves (intrinsic goals) and goals which are a means to another end (instrumental goals). Intrinsic goals have the following criteria [23]:

1. It must be possible to raise the level of the intrinsic goal while holding the levels of other metrics constant. That is, they must be at least partially independent of other goals.
2. Raising the level of the intrinsic goal is desirable. If it is not, it is probably an instrumental goal.

When these criteria are not fulfilled for a goal, it is likely to be an instrumental goal and indicative of a metric which may be a means to another end – usually an intrinsic goal is harder to quantify [23]. This is a crucial element in high-performance sport, as the balance of these is important for end-user engagement. For instance, any health programme that lowers performance outcomes is unlikely to resonate with athletes and coaches. In a public health setting, raising health can be seen as an intrinsic goal; however, in sport, this perspective may be held widely as health is seen as a vehicle for improved performance [65] or that injuries are only conceptualized as such once they limit performance [66]. In Australia, support personnel from sport science and medicine professions would be considered health services under the *Commonwealth of Australia Privacy Act*. Therefore, the main differentiator of a public health system from a sport health system can be conceptualized in the endpoint in which these services are provided. In high-performance sport, athletes seek to improve and maintain their physical and mental health as one of several strategies towards achieving overall strategies to reach their intrinsic goal or an equivalent performance outcome (e.g. to win, surpass a season or personal best). Therefore, athlete health may be conceptualized as both an intrinsic goal, in that being healthy is desirable, and an instrumental goal of performance outcomes (as performance cannot be conceptualized without considering athlete health during competition).

## How do the Four Essential Functions Integrate to Form a Health System?

**Fig. 2:**
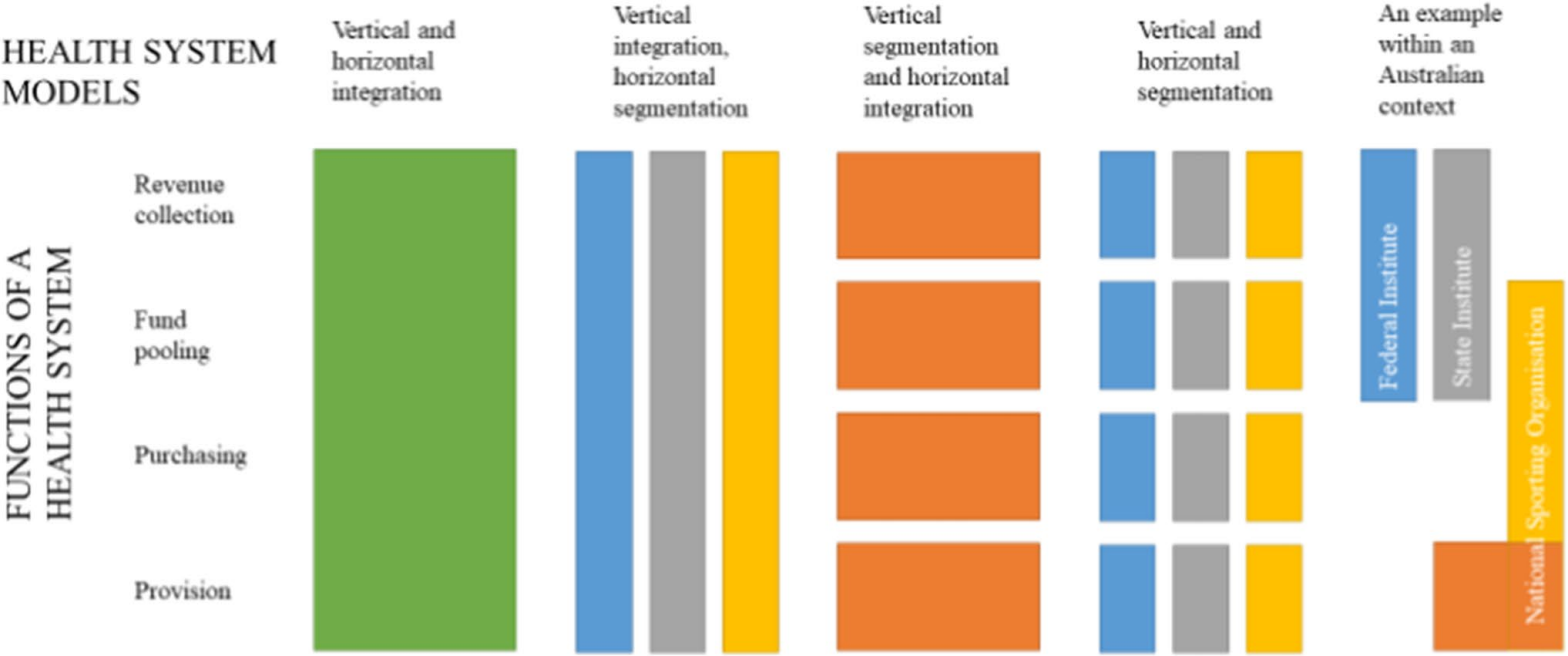
Health system models according to types of integration. Adapted from Murray and Frenk [23], with permission

Legend: Blue, federal institute; yellow, national sports organization; grey, state institute (or university/ college); orange, integrated provision by two or more organizations; green, hypothetical health system with universal provision by a single organization.

The four essential functions can be integrated, segmented or through a hybrid combination depending on which organization provides what function to which population. Below is the conceptual image [23] (adapted) to illustrate the four functions of a health system and how they may or may not integrate across three organisations in an Australian high-performance sport system (Fig. 2). When examining this model, the three organizations for consideration are the federal institute, state institute and NSO. These integrations are complex and unlikely to be mapped with such simplicity within the Australian high-performance sport system as indicated by a hypothetical scenario on the far right of Fig. 2.

## Key Challenges and Potential Solutions for Moving Towards a Health System Approach to Athlete Health Within High-Performance Sport

### Moving Towards an Integrated Performance System Inclusive of the Athlete Health Subsystem

The fundamental challenge for athlete health within a high-performance sport system is the balance between the need to achieve performance outcomes whilst ensuring the long-term health of athletes is protected in line with Hippocratic principles. The overarching system within high-performance sport has been referred to as ‘an integrated performance system’ [2]. On the surface, these two intrinsic goals may seem in opposition [1]. We suggest that to mitigate conflicts between health and performance systems, functions of the sport health system should be measured against its ability to safeguard athlete health within the overarching goal of improving athletes’ performance goals—in which athlete health is a necessary contributor towards sustainably achieving this outcome.

### Measuring and Monitoring of the Performance of the Sport Health System is Required

Performance in a health system setting is defined as the attainment of its goal(s) relative to its resources available [23]. Therefore, sport health system performance may be best conceptualized by considering its efficiency to meet individual athletes’ needs and the overarching objective of the integrated performance system. Future work should explore how sport health systems may be measured against their ability to improve, protect and maintain health relative to their available resources.

### Prevention and Treatment Programmes Must be Considered Within the Sport Health System’s Ability to Sustainably Implement Them

The challenges of implementing evidence-based interventions are well published [67]. However, we propose an additional barrier to implementation where interventions are developed outside the health system and have not considered contextual factors or change readiness in regards to organizational policy and process, culture, leadership support and resource allocation. We support and strongly advocate for interventions to be considered at a system-level rather than an individual or programme level [68] as resources are often at or beyond capacity.

### Health Data and Intelligence Management

In simple terms, health data are any data about one’s health or disability [69]. Many services provided to the athlete are health services [24] which aim at assessing, predicting, maintaining or improving the individual athlete’s physical, mental or psychological health or status. The data related to these health services are likely considered to be health information evidenced by the Australian Office of the Australian Information Commissioner[70] and therefore required to meet the privacy and associated legislation. There are many considerations for managing health information and we suggest further exploration of this topic is required. For brevity, five key considerations are presented:

1. How could/should data or other health information be used for the benefit of stakeholders (that is determining ‘value’ relative to ‘costs’/guide implementation/de-implementation of particular system elements to ensure desired effect is being achieved)?;
2. Technical/governance/socio-political challenges associated with accessing (or sharing) sensitive health-related information within and across organizations in federated systems affected by lack of interoperability in digital systems, incumbent legislation and local policies;
3. Sustainable resourcing to collect/collate/analyse the data to yield ‘intelligence’;
4. The extent of the social licence granted by stakeholders (athletes, health care providers, etc.) for system controllers to use data recorded for a primary purpose (e.g. data about an athlete’s health-related assessment or services accessed) to be used for a secondary purpose (e.g. making decisions about funding or not funding elements of the athlete’s health care system, measuring health system performance) or tertiary purpose (e.g. funding decisions related to athlete, programme, sport or in research projects); and
5. The levels of transparency around the use of data/intelligence, as well as other typical data and intelligence issues (privacy, confidentiality, security, etc.).

Moving forward, sports organizations and institutions should have health information policies to govern these areas.

## Conclusion

Safeguarding athlete health, in the interests of both sport and life goals, is the principal task of health services and systems in high-performance sport. The core challenge is striking a balance between the need to achieve performance outcomes whilst protecting current and long-term athlete health. Outside sport, health systems are recognized, monitored and evaluated. We offer a health system perspective to assist high-performance sport organizations to adopt these principles and seize the opportunity to optimise both athlete health and performance concurrently. We welcome further debate and perspectives on this topic.

## Declarations

### Availability of data and material

Data sharing not applicable to this article as no datasets were generated or analysed during the current study.
